# 7Hsp70 serum levels in pet dogs—a potential diagnostic biomarker for spontaneous round cell tumors

**DOI:** 10.1007/s12192-019-01024-9

**Published:** 2019-08-03

**Authors:** Lukas Salvermoser, Susann Dressel, Sarah Schleißheimer, Stefan Stangl, Christopher Diederichs, Melanie Wergin, Carla Rohrer Bley, Bernhard Haller, Gabriele Multhoff

**Affiliations:** 1 Radiation Immuno-Oncology Group, Center for Translational Cancer Research Technische Universität München (TranslaTUM), Klinikum rechts der Isar, Technische Universität München (TUM), Einstein Str. 25, 81675 Munich, Germany; 2 Division of Radiation Oncology, Vetsuisse Faculty, University of Zurich, Winterthurer Str. 258c, CH-8057 Zurich, Switzerland; 3 Medizinische Kleintierklinik, Ludwig-Maximilian-Universität München, Veterinär Str. 13, 80539 Munich, Germany; 4 Institute for Medical Informatics, Statistics and Epidemiology, Technische Universität München, Klinikum rechts der Isar, Ismaninger Str. 22, 81675 Munich, Germany

**Keywords:** lipHsp70 ELISA, Canine Hsp70, Tumor biomarker, Round cell tumor, Mast cell tumor

## Abstract

The concentration of circulating heat shock protein 70 (Hsp70) was measured in liquid biopsies of canine tumor patients as a potential biomarker. Compared with rodent tumor models, spontaneously occurring tumors in pet dogs reflect the clinical situation of human patients better, as dogs cohabitate with their owners in the same environment, reach a much older age than rodents, can provide blood samples much more frequently, and receive up-to-date medical care and, similar to humans, their tumors show a high genetic heterogeneity. Due to the species-specific sequence homology of human and canine Hsp70, two human enzyme-linked immunosorbent assay (ELISA) systems (R&D and lipHsp70) were used to measure canine Hsp70 concentrations in serum and plasma. In general, higher Hsp70 concentrations were found in serum compared with plasma samples of dogs, and the lipHsp70 ELISA detected higher peak concentrations of Hsp70 in a broader range than the R&D ELISA. Compared with a tumor-free control group, serum Hsp70 concentrations were higher in tumor-bearing dogs, irrespective of breed, age, body weight, and gender. A sub-classification of the different tumors according to their cytological characteristics revealed significantly elevated Hsp70 serum concentrations in dogs with round cell tumors (*p* < 0.01), a heterogeneous group of malignancies with hematopoietic origin such as mast cells, plasma cells, lymphocytes, histiocytes, and melanomas. Future studies with larger patient cohorts and well-defined tumor sizes are necessary to elucidate the role of serum Hsp70 as a biomarker for tumor detection and monitoring of outcome in pet animals.

## Introduction

Members of the different heat shock protein (HSP) families are highly conserved in evolution with a high sequence homology among different species (Hartl 1996; Daugaard et al. 2007; Shalgi et al. 2014). According to their molecular weights, HSPs are subdivided into large (HSP110, HSP90, HSP70, HSP60) and small (HSP10, HSP25, HSP27) families (Horvath et al. 2008). The major stress-inducible Hsp70 with a molecular weight of approximately 70 kDa forms multichaperone complexes together with Hsp40 and Hsp90 and the co-chaperones HiP and HoP (Wegele et al. 2004; Qiu et al. 2006). In contrast to most proteins whose expression is downregulated upon stress, the synthesis of HSPs, and especially that of Hsp70, is highly upregulated (Daugaard et al. 2007). Apart from heat stress, a large variety of other physical and chemical stress stimuli, as well as physiological processes such as proliferation, differentiation, and antigen presentation, can also trigger the synthesis of HSPs (Lindquist and Craig 1988; Hartl 1996; Whitley et al. 1999). Following the translocation of the trimerized heat shock factor into the nucleus and binding to the heat shock element in the promotor region, the expression of HSPs is induced (Akerfelt et al. 2010).

Members of the HSP70 family are found in nearly all subcellular compartments, including the cytosol, nucleus, endoplasmic reticulum, mitochondria, lysosomes, and endosomes (Radons and Multhoff 2005). As molecular chaperones, intracellular HSP70s are involved in the correct folding of unfolded proteins, proteasomal degradation, transport across membranes (Ostermann et al. 1990), prevention of protein aggregation, apoptosis after stress, and cell cycle regulation (Hunt and Morimoto 1985; Lindquist and Craig 1988; Hartl 1996; Selvarajah et al. 2013).

It is well established that, compared with normal cells, tumor cells overexpress Hsp70 even under non-stress conditions (Hantschel et al. 2000; Ciocca and Calderwood 2005; Rohde et al. 2005). Although Hsp70 is primarily a cytosolic protein, our group was the first to demonstrate membrane localization of Hsp70 on a large variety of human and mouse tumor cell types (Hantschel et al. 2000; Multhoff et al. 1995; Stangl et al. 2011). Moreover, membrane Hsp70-positive tumor cells have been found to actively release Hsp70 in lipid microvesicles (Cordonnier et al. 2017) with molecular characteristics of exosomes (Gastpar et al. 2005). Free Hsp70 originating from dying cells and exosomal Hsp70 which is actively released by viable tumor cells can be quantified in the blood of patients with tumors by using the novel lipHsp70 ELISA (Breuninger et al. 2014). This ELISA is based on the cmHsp70.1 mouse IgG1 monoclonal antibody (mAb) detecting an 8-mer epitope of Hsp70 in the C-terminal oligomerization domain that is exposed on the surface of tumor cells and tumor-derived exosomes (Stangl et al. 2011; Breuninger et al. 2014). Consequently, elevated Hsp70 serum and plasma levels could be correlated with the viable tumor mass in patients with non-small cell lung carcinoma (NSCLC) before and after radiochemotherapy (Gunther et al. 2015).

In the present study, we addressed the question whether circulating exosomal Hsp70 determined by the lipHsp70 ELISA could also provide a useful tumor biomarker in liquid biopsies for companion animals. In pet dogs, elevated intracellular Hsp70 levels were reported in osteosarcoma (Asling et al. 2016), mammary (Romanucci et al. 2006; Kumaraguruparan et al. 2006), and transmissible venereal tumors (Chu et al. 2001), as determined by Western blot analysis, and immunohistochemical staining. Herein, we examined Hsp70 plasma and serum levels in dogs with different tumors and in tumor-free control animals. According to their cytological characteristics, tumors in dogs were classified in three main groups: mesenchymal, epithelial, and round cell (Withrow et al. 2012; Villiers et al. 2016; Raskin and Meyer 2016). Canine tumors derived from the mesenchyme comprise degenerations of osseous and soft tissues, different types of sarcoma, and neoplasms of blood vessels. Tumors of epithelial origin included cancers of glands and epithelial surfaces such as the skin, respiratory, and gastrointestinal tract. Canine round cell tumors mainly consist of tumors of hematopoietic origin such as mast cells, plasma cells, lymphocytes, and histiocytes and also include melanomas according to their cytological classification (Raskin and Meyer 2016).

## Material and methods

### Cell culture of canine tumor cell lines

Two canine tumor cell lines (K9STS, mesenchymal tumor; K9MM2, round cell tumor) derived from primary tumors were kindly provided by Professor Dr. Rohrer Bley. K9STS cells were cultured at 37 °C under 5% CO_2_ in a humidified atmosphere in RPMI 1640 medium (R8758, Sigma Aldrich, St. Louis, MO, USA) supplemented with 10% FCS (F7524, Sigma Aldrich, St. Louis, MO, USA), 1% HEPES (15630-056, Gibco, Thermo Scientific, Rockford, IL, USA), 1% l-glutamine (G7513, Sigma Aldrich, St. Louis, MO, USA), 1% MEM non-essential amino acid (NEAA) (11140-050, Gibco, Thermo Scientific, Rockford, IL, USA), 1% sodium pyruvate (S8636, Sigma Aldrich, St. Louis, MO, USA), and 1% penicillin-streptomycin (P0781, Sigma Aldrich, St. Louis, MO, USA). K9MM2 cells were cultured in high glucose Dulbecco’s Modified Eagle Medium (DMEM) (D6429, Sigma Aldrich, St. Louis, MO, USA) supplemented with 10% FCS, 1% HEPES, and 1% penicillin-streptomycin at 37 °C under 5% CO_2_ in a humidified atmosphere. Cells were regularly passaged every second day and used for experiments in the exponential growth phase. Cell lines were tested negative for mycoplasma contamination.

### Western blot

K9STS and K9MM2 cells (2 × 10^6^) were lysed in radioimmunoprecipitation assay (RIPA) buffer for 20 min on ice after washing three times in PBS. Cell lysates were centrifuged at 12,000*g* for 20 min at 4 °C, and supernatant was transferred into Eppendorf tubes. Protein concentrations were determined using the BCA Protein Assay Kit (23225, Pierce, Thermo Scientific, Rockford, IL, USA). Thirty micrograms of cytosolic protein from each cell line was loaded on a 10% SDS-PAGE. Human recombinant Hsp70 protein (32.5 ng) was used as an internal control.

A nitrocellulose membrane (10600002, GE Healthcare, Wauwatosa, WI, USA) was used for blotting. Membranes were blocked using 5% skim milk in TBST for 1 h at room temperature. Primary antibodies, anti-Hsp70: cmHsp70.1 (multimmune, Munich, Germany, 1:1000), and anti ß-actin: A2228 (Sigma Aldrich, St. Louis, MO, USA; 1:10000) were diluted in 5% skim milk/TBST and incubated at 4 °C overnight. Membranes were washed three times with TBST. The secondary, horseradish-peroxidase (HRP)–conjugated antibody (P0260, Dako, Agilent, Santa Clara, CA, USA) was diluted 1:1,000 in 5% skim milk/TBST and incubated for 1 h at room temperature. Membranes were washed three times for 15 min at room temperature using TBST and subjected to ECL Western blotting substrate (32106, Pierce, Thermo Scientific, Rockford, IL, USA) for 30 s in the dark before exposure (1708370, ChemiDoc Touch Imaging System, Biorad Laboratories, Hercules, CA, USA).

### lipHsp70 ELISA

For measuring the Hsp70 content in canine plasma and serum samples, the lipHsp70 ELISA that was established for human blood samples to quantify free and liposomal Hsp70 in the circulation was used. For the lipHsp70 ELISA, 96-well MaxiSorp Nunc-Immuno plates (442404, Thermo, Rochester, NY, USA) were coated overnight at room temperature with 2 μg/ml rabbit polyclonal antibody (Davids, Biotechnologie, Regensburg, Germany) directed against human recombinant Hsp70 in sodium carbonate buffer (0.1 M sodium carbonate, 0.1 M sodium hydrogen carbonate, pH 9.6). After washing three times with PBS (D8537, Sigma Aldrich, St. Louis, MO, USA)/0.05% Tween-20 (655205, Merck, Darmstadt, Germany), the assay was blocked with 2% skim milk (T145.2, Carl Roth, Karlsruhe, Germany) in PBS for 1.5 h at 27 °C. A dilution of 1:5 of the canine samples in CrossDown Buffer (A6485, Applichem, Darmstadt, Germany) appeared to be optimal for the ELISA assay. Following another washing step, 100 μl of the diluted samples was added to the wells for 2 h at 27 °C. Then, the plates were washed in PBS and incubated with 3 μg/ml biotinylated mouse anti-human cmHsp70.1 mAb (multimmune, Munich, Germany) in 2% skim milk in PBS for 2 h at 27 °C. Finally, after another washing step, 0.2 μg/ml HRP-conjugated streptavidin (DRG, Marburg, Germany) in 1% bovine serum albumin (A7030, Sigma Aldrich, St. Louis, MO, USA) was added for 1 hat 27 °C. Binding was quantified by adding the substrate reagent (DYC1663E, R&D Systems, Minneapolis, MN, USA) for 30 min at 27 °C. The absorbance was measured at a wavelength of 450 nm, corrected by the absorbance at 570 nm using a Microplate Reader (ELx800, BioTek, Winooski, VT, USA). A standard curve using eight concentrations of recombinant Hsp70, ranging from 0 to 50 ng/ml diluted in CrossDown Buffer, was included in each ELISA test. The blank was determined by measuring the absorbance of PBS diluted 1:5 in CrossDown Buffer. For all concentrations below the established limit of detection of 0.31 ng/ml (Breuninger et al. 2014) measured with lipHsp70 ELISA, 0.31 ng/ml was valued in statistical analysis.

### R&D systems Hsp70 ELISA

As a control, all serum samples were also measured with the commercial DuoSet® IC Human/Mouse/Rat total Hsp70 ELISA (DYC1663E, R&D Systems, Minneapolis, MN, USA). The DuoSet IC Human/Mouse/Rat total Hsp70 ELISA was applied following the manufacturer’s instructions.

### Statistics

For each individual animal, Hsp70 was tested in at least three experiments (maximum 12 experiments). Mean values of all evaluable values were calculated for each individual animal and those mean values were considered for statistical analysis. For animals that had no single measure above the minimum detection level, the value 0.31 ng/ml was used. As distribution of Hsp70 data were skewed, median values and quartiles are presented as median (1st quartile–3rd quartile). For comparison of independent samples, Wilcoxon rank-sum tests were performed. The Wilcoxon signed-rank test was used to compare paired data. For the receiver operating characteristics (ROC) analyses, assessing whether Hsp70 levels can be used to discriminate healthy from diseased dogs, only healthy animals and animals suffering from disease of interest were included. For each analysis, the area under the ROC curve (AUC), the Hsp70 cut-off value giving the highest sum of sensitivity and specificity, and the corresponding sensitivity and specificity are presented. All statistical tests were performed two-sided and a significance level of *α* = 5% was used.

## Results

### cmHsp70.1 monoclonal antibody detected canine Hsp70

Recently, our group has developed the lipHsp70 ELISA to quantify liposomal and free Hsp70 in serum and plasma of human donors (Breuninger et al. 2014). By comparing Hsp70 levels of patients with tumors with an age- and gender-matched control cohort, we demonstrated that patients with tumors revealed significantly higher Hsp70 concentrations in the blood than healthy human volunteers. Herein, we addressed whether the lipHsp70 ELISA could be used to detect Hsp70 in the serum and plasma of pet dogs with spontaneous tumors.

**Fig. 1:**
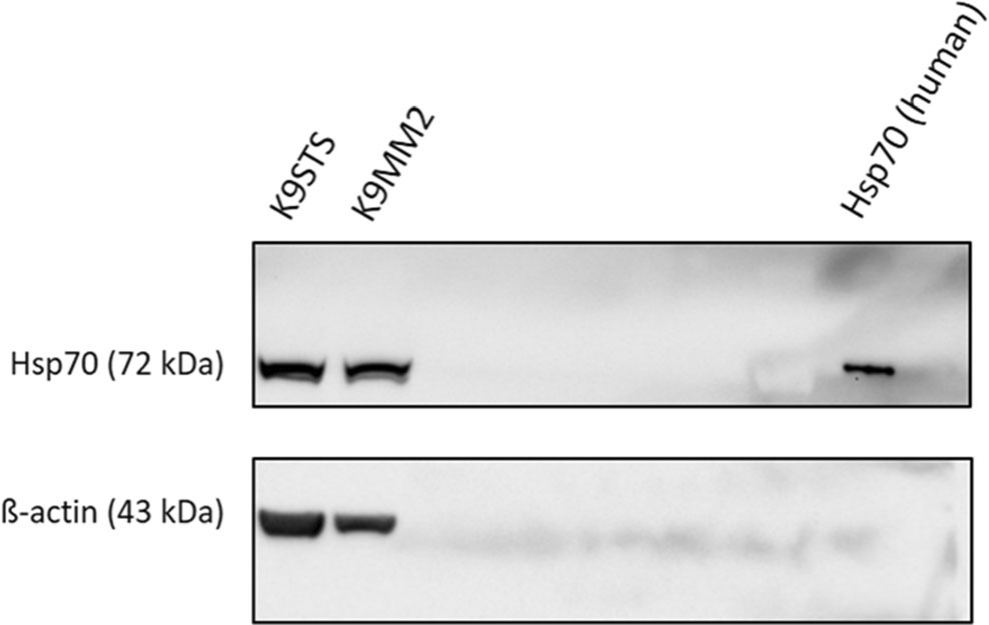
Western blot analysis of cell lysates of canine soft tissue sarcoma (K9STS) and malignant melanoma (K9MM2). Lane 1: cell lysate of K9STS. Lane 2: cell lysate of K9MM2 cells (30 μg protein each). Lane 3: human recombinant Hsp70 (32.5 ng) as a positive control. ß-Actin served as a loading control

As mentioned above, the Hsp70 protein is highly conserved across different species, including humans, mice, rats, and dogs. A comparison of the 8-mer epitope (N-L-L-G-R-F-E-L) that is recognized by the cmHsp70.1 mAb (Stangl et al. 2011) showed that this protein sequence is identical in human and canine Hsp70 (UniProt database: human Hsp70: P0DMV8, canine Hsp70: Q7YQC6). Furthermore, Western blot analysis of cell lysates in canine soft tissue sarcoma (K9STS, mesenchymal tumor) and malignant melanoma (K9MM2, round cell tumor) revealed a specific band at 72 kDa using cmHsp70.1 antibody (Fig. 1). The 43-kDa ß-actin band served as a loading control (Fig. 1), and human recombinant Hsp70 protein was subjected as a molecular weight control (Fig. 1). These data indicated that cmHsp70.1 mAb is able to detect human as well as canine Hsp70.

### Hsp70 serum levels were higher in canine serum compared with plasma

To determine whether Hsp70 could be detected in canine blood using the human lipHsp70 ELISA, serum (S) and plasma (P) samples were collected from healthy control (S: *n* = 38, P: *n* = 25) and tumor-bearing dogs (S: *n* = 74, P: *n* = 42). The study was carried out in strict accordance with the recommendations and the protocol approved by the Animal Ethics Council of the Canton Zurich, Switzerland and the Ludwig-Maximilians University Munich, Germany.

**Fig. 2:**
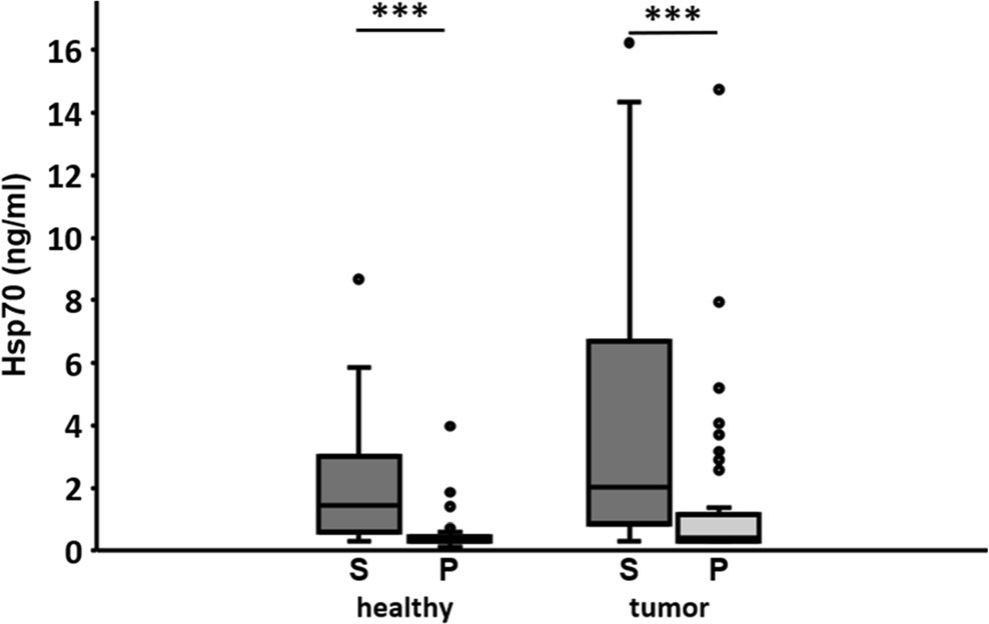
Comparison of the Hsp70 concentrations in serum and plasma as determined by the lipHsp70 ELISA. Serum (S: *n* = 112) and plasma (P: *n* = 67) samples were taken from healthy control (S: *n* = 38; P: *n* = 25) and tumor-bearing (S: *n* = 74; P: n = 42) pet dogs. Lines inside the box plots show the median value, upper and lower boundaries indicate the 25th and 75th percentile, and whiskers indicate highest and lowest value within 1.5 IQR, respectively. Not all outliers are shown. ***p < 0.001

Serum and plasma samples were measured with the lipHsp70 ELISA using the protocol established for human blood samples (Breuninger et al. 2014). As Fig. 2 shows, Hsp70 concentrations were higher in serum and plasma samples of canines with tumors (median S: 1.98 (0.84–6.26) ng/ml; median P: 0.43 (0.31–1.08) ng/ml) compared with healthy control animals (median S: 1.43 (0.68–2.97) ng/ml; median P: 0.31 (0.31–0.40) ng/ml). In contrast to human samples, Hsp70 values were significantly higher in serum compared with plasma samples in both animal groups (*p* < 0.001) (Fig. 2).

**Fig. 3:**
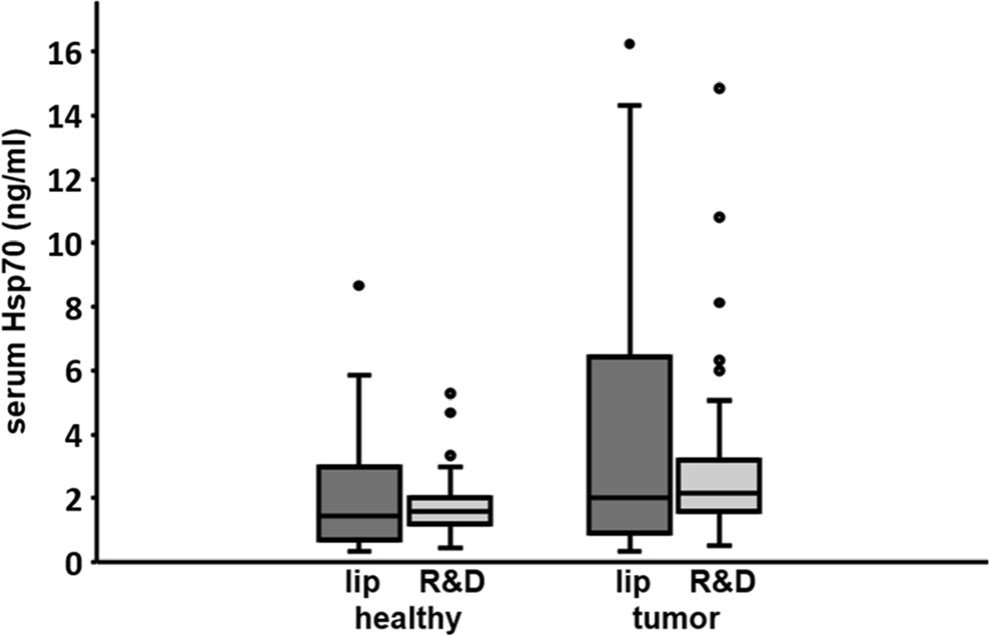
Comparison of the Hsp70 concentrations in serum as determined by the lipHsp70 and R&D ELISA. Serum samples of 38 healthy and 74 tumor-bearing canines were measured with lipHsp70 ELISA in quadruplicates and with R&D ELISA in duplicates. Lines inside the box plots show the median value, upper and lower boundaries indicate the 25th and 75th percentile, and whiskers indicate highest and lowest value within 1.5 IQR, respectively. Not all outliers are shown

In human serum and plasma samples, the cmHsp70.1 mAb used as a detection antibody in the lipHsp70 ELISA, detects free and lipid-bound exosomal Hsp70, which was actively released by viable tumor cells (Stangl et al. 2011; Gastpar et al. 2005). Therefore, the lipHsp70 ELISA detected higher Hsp70 concentrations in the blood of humans than the commercially available R&D ELISA. In canine serum samples, the range of the Hsp70 concentrations in control (median lip control: 1.43 (0.68–2.97) ng/ml) and tumor-bearing animals (median lip tumor-bearing: 1.98 (0.84–6.26) ng/ml) measured with the lipHsp70 ELISA was larger than that measured with the R&D ELISA (median R&D control: 1.57 (1.20–2.02) ng/ml; median R&D tumor-bearing: 2.12 (1.55–3.18) ng/ml (Fig. 3).

### Food intake and up to three freezing/thawing cycles did not affect Hsp70 serum levels in dogs

**Fig. 4:**
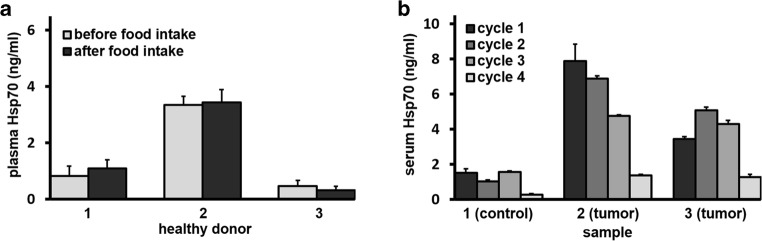
Influence of external interference factors on Hsp70 serum concentrations as determined by the lipHsp70 ELISA. **a** Serum samples were taken from three healthy dogs with a different Hsp70 concentrations before (gray) and 2 h after intake (black bars) of a high-fat diet. Determination of Hsp70 concentrations revealed no significant differences before and after food intake for each dog. **b** Serum of both control and tumor-bearing dogs was subjected to up to eight freezing/thawing cycles (only the first four cycles shown). The Hsp70 concentration was determined after each cycle using the lipHsp70 ELISA. Levels remained nearly unchanged until the third freezing/thawing cycle. Thereafter, a drop in the Hsp70 concentrations was detectable (data not shown)

Potential effects of external interference factors that could impact Hsp70 serum levels of dogs were assessed. Food intake and repeated freezing/thawing cycles of the serum samples were tested regarding the detection of free and lipid-bound Hsp70 by the lipHsp70 ELISA. Serum was collected from healthy dogs that differed in their basal Hsp70 concentrations (dog 1: 0.8 ng/ml; dog 2: 3.3 ng/ml; dog 3: 0.5 ng/ml) before and 2 h after intake of a high-fat diet. The quantity of given food was adjusted to the metabolic body mass of each animal to ensure that the calorie intake per kilogram metabolic bodyweight was identical for each animal. The amount of food was equal to one-third of each dog’s daily food ration. As Fig. 4a shows, food intake did not influence the Hsp70 serum levels within the given time period. Another test was performed to study the influence of repeated freezing/thawing of the serum samples on the Hsp70 content that was detectable by the lipHsp70 ELISA. Serum samples of dogs that differed in their basal Hsp70 serum concentrations (dog 1: 1.5 ng/ml; dog 2: 7.9 ng/ml; dog 3: 3.5 ng/ml) were subjected to eight repeated freezing/thawing cycles, and Hsp70 concentrations were measured after each cycle. Our results revealed that Hsp70 concentrations measured by the lipHsp70 ELISA remained almost stable for up to three repeated freezing/thawing cycles (Fig. 4b); however, thereafter, a drop of the Hsp70 concentrations was detectable in all analyzed samples (data not shown).

### Hsp70 serum levels were significantly elevated in dogs with round cell tumors

As Table 1 summarizes, the control group consisted of 38 healthy dogs of different ages and breeds. The animals were admitted to the veterinary clinic for routine examinations and procedures such as vaccination, hip X-ray, or sterilization. None of the animals showed any signs of infection or inflammation at the time of blood collection. Hsp70 serum levels did not show any correlation with age, weight, gender, breed, and sterilization status of the dogs (data not shown).

**Table 1:** Breed, age, weight, and gender of healthy control dogs

No.	Breed	Age	Weight (kg)	Gender
1	Cross-breed	2	16	Female
2	French Bulldog	8	10	Female
3	Labrador Retriever	2	27	Male
4	Irish Terrier	6	15	Female
5	German Shepherd	2	38	Male
6	Australian Cattle	5	20	Male
7	Cross-breed	12	15	Female
8	Hovawart	8	43	Male
9	Golden Retriever	5	30	Male
10	Beagle	3	12	Male
11	Siberian Husky	1	14	Female
12	Labrador Retriever	5	32	Male
13	Labrador Retriever	9	37	Male
14	Chihuahua	9	3	Female
15	Flat-coated Retriever	8	33	Female
16	Labrador Retriever	10	35	Male
17	Beagle	4	13	Female
18	Beagle	4	12	Female
19	Beagle	4	12	Female
20	Flat-coated Retriever	6	33	Male
21	Cross-breed	14	8	Female
22	Dalmatian	5	27	Male
23	Cross-breed	4	20	Female
24	Cross-breed	8	32	Male
25	Flat-coated Retriever	8	28	Female
26	Flat-coated Retriever	5	28	Female
27	Flat-coated Retriever	1	30	Female
28	Beagle	4	17	Male
29	Beagle	4	17	Female
30	Small Munsterlander	5	17	Female
31	Dachshund	10	8	Female
32	Rottweiler	1	38	Female
33	Cross-breed	12	21	Male
34	Nova Scotia Duck Tolling Retriever	1	19	Male
35	Jack Russell Terrier	9	9	Male
36	Lagotto Romagnolo	2	14	Female
37	French Bulldog	8	10	Male
38	German Shepherd	5	30	Male

The tumor cohort consisted of 74 animals with cytological and histopathological confirmed neoplastic diseases. All tumors spontaneously occurred, and all dogs lived in a non-sterile environment.

**Fig. 5:**
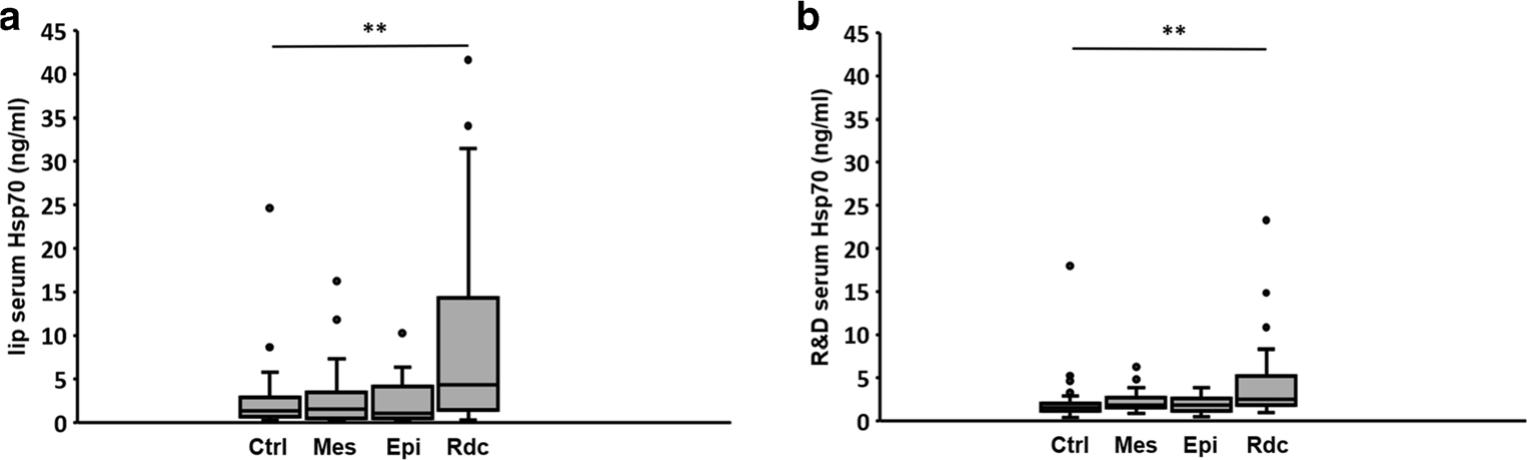
Comparative analysis of Hsp70 serum concentrations as determined by the lipHsp70 (**a**) and R&D (**b**) ELISA in control and tumor-bearing pet dogs. Serum samples were taken from a total of 112 dogs of different breeds: healthy control dogs (Ctrl: *n* = 38), dogs with mesenchymal tumors (Mes:, *n* = 27), dogs with epithelial tumors (Epi: *n* = 22), and dogs with round cell tumors (Rdc: *n* = 25). Tables 1, 2, 3, and 4 summarize characteristics of the dog patients. Lines inside the box plots show the median value, upper and lower boundaries indicate the 25th and 75th percentile, and whiskers indicate highest and lowest value within 1.5 IQR, respectively. **p < 0.01

According to their cytological characteristics (Tables 2, 3, and 4), the tumors could be sub-classified as mesenchymal, epithelial, and round cell. Hsp70 serum concentration was measured in healthy dogs (*n* = 38) and canines with mesenchymal (*n* = 27), epithelial (*n* = 22), and round cell tumors (*n* = 25) using the lipHsp70 and R&D ELISA. As Fig. 5 shows, the Hsp70 serum concentrations of dogs with round cell tumors were significantly higher (median lip: 4.37 (1.44–14.32) ng/ml; median R&D: 2.55 (1.89–5.28) ng/ml) compared with the control group (median lip: 1.43 (0.68–2.97) ng/ml; median R&D: 1.57 (1.20–2.02) ng/ml) (*p* < 0.01) (Fig. 5a, b). In contrast, the concentrations of Hsp70 in dogs with mesenchymal (median lip: 1.57 (0.56–3.55) ng/ml; median R&D: 1.90 (1.59–2.77) ng/ml) and epithelial tumors (median lip: 1.11 (0.52–4.18) ng/ml; median R&D: 1.83 (1.21–2.62) ng/ml) were not significantly different to that of healthy animals (Fig. 5a, b).

**Table 2:** Characteristics of dogs diagnosed with mesenchymal tumors

#	Diagnosis	Tumor location	Extension	Breed	Age	Weight (kg)	Gender
1	Hemangiopericytoma	Tarsus	Local	Cross-breed	11	33	Female
2	Hemangiopericytoma	Elbow	Local	Irish Red and White Setter	10	35	Female
3	Hemangiopericytoma	Carpus	Local	Boxer	11	30	Female
4	Hemangiopericytoma	Thigh	Local	Labrador Retriever	11	37	Male
5	Hemangiopericytoma	Forepaw	Local	Cross-breed	10	8	Female
6	Hemangiopericytoma	Elbow	Local	Irish Red and White Setter	11	38	Female
7	Hemangiopericytoma	Elbow	Systemic	Cross-breed	8	41	Male
8	Hemangiopericytoma	Elbow, nasal	Systemic	Malinois	12	26	Female
9	Hemangiosarcoma	Inguinal	Local	British Bulldog	6	29	Male
10	Spindle cell sarcoma, Hemangiosarcoma	Perianal, thigh	Systemic	Schnauzer	11	55	Female
11	Spindle cell sarcoma	Tongue	Local	Australian Shepherd	8	33	Female
12	Spindle cell sarcoma	Foreleg	Local	Borzoi	9	50	Male
13	Spindle cell sarcoma	Tarsus	Local	Flat-coated Retriever	10	27	Female
14	Spindle cell sarcoma	Soft palate	Local	Labrador Retriever	7	35	Male
15	Spindle cell sarcoma	Neck	Local	American Bulldog	12	34	Female
16	Soft tissue sarcoma	Pelvis	Local	Labrador Retriever	9	24	Female
17	Soft tissue sarcoma	Tarsal bones	Local	Golden Retriever	9	34	Male
18	Soft tissue sarcoma	Perianal	Local	Labrador Retriever	12	35	Male
19	Fibrosarcoma	Maxilla	Local	Magyar Vizsla	12	29	Male
20	Fibrosarcoma	Hindleg	Local	Bernese Mountain	1	42	Male
21	Fibrosarcoma	Mandible thorax	Systemic	Golden Retriever	9	25	Female
22	Osteosarcoma	Humerus	Local	Cross-breed	11	19	Female
23	Osteosarcoma	Humerus	Local	Hovawart	12	31	Female
24	Osteosarcoma	Femur	Local	Labrador, Retriever	13	29	Female
25	Osteosarcoma	Radius	Local	Leonberger	5	48	Female
26	Osteosarcoma	Humerus	Local	Rottweiler	10	39	Female
27	Osteosarcoma	Humerus heart	Systemic	Labrador Retriever	9	29	Female

**Table 3:** Characteristics of dogs diagnosed with epithelial tumors

#	Diagnosis	Tumor location	Extension	Breed	Age	Weight (kg)	Gender
1	Anal gland carcinoma	Anal sac	Local	Cross-breed	7	37	Male
2	Anal gland carcinoma	Anal sac	Local	Cross-breed	9	25	Male
3	Anal gland carcinoma	Anal sac	Local	Border Collie	10	26	Male
4	Anal gland carcinoma	Anal sac	Local	Labrador Retriever	10	27	Male
5	Anal gland carcinoma	Anal sac	Local	Anatolian Shepherd	10	26	Male
6	Anal gland carcinoma	Anal sac	Systemic	Australian Shepherd	12	23	Male
7	Adenocarcinoma	Nasal cavity	Local	Pug	9	11	Female
8	Adenocarcinoma	Nasal cavity	Local	Fox Terrier	8	10	Male
9	Adenocarcinoma	Nasal cavity	Local	Cross-breed	12	17	Male
10	Carcinoma	Nasal cavity	Local	Cross-breed	11	24	Male
11	Carcinoma	Nasal cavity	Local	Golden Retriever	13	38	Male
12	Carcinoma	Nasal cavity	Systemic	Springer Spaniel	14	21	Male
13	Carcinoma	Nasal cavity neck	Local	Golden Retriever	13	31	Female
14	Thyroid carcinoma	Neck	Local	Kerry Blue Terrier	13	16	Female
15	Thyroid carcinoma	Neck	Local	West Highland White Terrier	6	10	Male
16	Thyroid carcinoma	Neck	Local	Labrador Retriever	12	37	Male
17	Salivary gland carcinoma	Neck	Local	Portuguese Podenco	11	7	Female
18	Prostate carcinoma	Prostate	Local	Bergamasco Shepherd	11	35	Male
19	Squamous cell carcinoma	Tongue	Local	Appenzeller Sennenhund	13	50	Female
20	Squamous cell carcinoma	Toe	Systemic	Labrador Retriever	12	21	Male
21	Basal cell carcinoma	Nose	Local	Parson Russell Terrier	8	27	Male
22	Leydig cell tumor	Testicle	Local	Border Terrier	12	31	Male

**Table 4:** Characteristics of dogs diagnosed with round cell tumors

#	Diagnosis	Tumor location	Extension	Breed	Age	Weight (kg)	Gender
1	Mast cell tumor	Nasal cavity	Local	Berger de Pyrenees	13	7	Female
2	Mast cell tumor	Nasal cavity	Local	Magyar Vizsla	8	27	Male
3	Mast cell tumor	Skin	Local	Labrador Retriever	12	31	Male
4	Mast cell tumor	Rhinarium	Local	Jack Russel Terrier	12	12	Male
5	Mast cell tumor	Abdomen	Local	Schnauzer	10	29	Female
6	Mast cell tumor	Skin	Local	Bernese Mountain	10	41	Female
7	Mast cell tumor	Thorax, thigh, ear	Systemic	Pug	9	7	Female
8	Mast cell tumor	Shoulder, thorax, tarsus	Systemic	Golden Retriever	8	25	Female
9	Mast cell tumor	Ear, scapula, thorax, thigh	Systemic	Greater Swiss Mountain	7	46	Female
10	Mast cell tumor	Shoulder	Local	Cross-breed	12	27	Female
11	Mast cell tumor	Nose	Local	Boxer	7	36	Male
12	Mast cell tumor	Thoracic wall	Systemic	Labrador Retriever	8	25	Male
13	T cell lymphoma	Mandible, lip	Local	Maltese	13	3	Male
14	T cell lymphoma	Jejunum, liver, spleen	Local	Greyhound	9	5	Male
15	Plasmocytoma	Rectum	Local	Cross-breed	13	30	Male
16	Follicular lymphoma	Mandibular lymph nodes	Local	Maltese	7	4	Male
17	B cell lymphoma	Liver, spleen	Systemic	Cross-breed	14	24	Female
18	B cell lymphoma	Multicentric	Systemic	Golden Retriever	7	39	Male
19	Amelanotic melanoma	Lip	Local	Golden Retriever	15	27	Female
20	Amelanotic melanoma	Mandible	Local	Hovawart	12	33	Female
21	Amelanotic melanoma	Gingiva, lung	Systemic	Poodle	9	22	Male
22	Malignant melanoma	Mandible, oral mucosa	Local	Boxer	15	30	Female
23	Malignant melanoma	Maxilla, orbital cavity	Local	Prague Ratter	14	3	Male
24	Malignant histiocytosis	Popliteal lymph node, liver, lung	Systemic	Bernese Mountain	4	47	Male
25	Malignant histiocytosis	Humerus, ulna, lung, liver, spleen	Systemic	Rottweiler	5	48	Male

To investigate potential variations in serum Hsp70 concentrations that might depend on the time of the admission of the dog to the clinic, serum samples of dogs with round cell tumors were collected over a time period of 2 years. Hsp70 serum concentrations of dogs with round cell tumors collected in the first year (median cohort 1: 4.18 (1.33–14.26) ng/ml) did not differ significantly in their distribution from samples collected in the second year (median cohort 2: 6.97 (2.35–26.99) ng/ml). The values of both cohorts differed significantly from those of control animals (median: 1.43 (0.68–2.97) ng/ml).

**Fig. 6:**
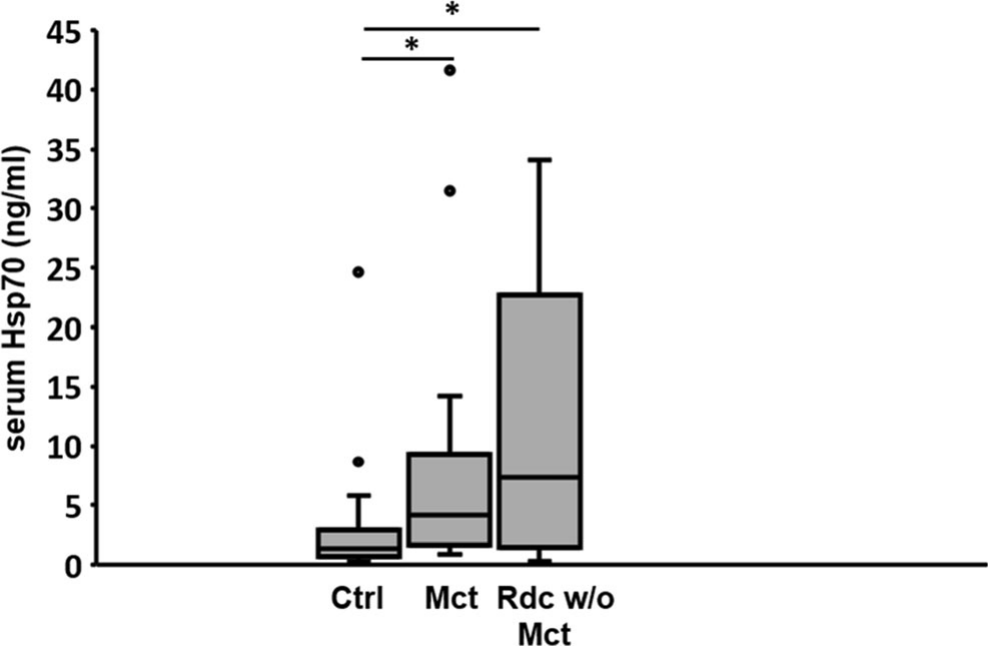
Comparative analysis of Hsp70 serum concentrations as determined by the lipHsp70 ELISA in dogs with mast cell tumors (Mct) and other round cell tumors. Within the group of patients with round cell tumors, dogs with mast cell tumors (Mct: *n* = 12) showed the highest Hsp70 serum concentrations followed by all other round cell tumor subtypes (Rdc without Mct: *n* = 13). Hsp70 serum levels of Mct and other Rdc were significantly higher than those of healthy controls. Lines inside the box plots show the median value, upper and lower boundaries indicate the 25th and 75th percentile, and whiskers indicate highest and lowest value within 1.5 IQR, respectively. *p < 0.05

The ROC analysis comparing the round cell tumor group with healthy dogs provided an AUC of 72%. A cut-off value of 2.33 ng/ml (sensitivity 64%, specificity 66%) was used as a threshold for Hsp70 serum content as a possible tumor biomarker in canine round cell tumors. A further subgroup analysis of dogs with round cell tumors revealed that canine patients with mast cell tumors had significantly higher Hsp70 serum concentration (median: 4.18 (1.71–9.26) ng/ml) compared with control animals (median: 1.43 (0.68–2.97) ng/ml) (Fig. 6). For the mast cell tumor subgroup, the AUC was 74%. An Hsp70 serum concentration of 2.36 ng/ml (sensitivity 67%, specificity 66%) was determined as a cut-off value to detect canine mast cell tumors. The mixed group of canine patients suffering from round cell tumors other than mast cell tumors also significantly (*p* < 0.05) differed from control animals (Fig. 6). A further subgroup analysis of dogs with melanoma revealed elevated Hsp70 serum levels compared with control animals as well; however, due to the relatively small sample size (*n* = 5), the values did not reach statistical significance (*p* = 0.07, the Wilcoxon rank-sum test).

## Discussion

In humans, the value of circulating exosomal and free Hsp70 as a diagnostic biomarker in serum and plasma has already been demonstrated for different tumor entities (Breuninger et al. 2014). Presently, there are two main pathways for Hsp70 to be released into the circulation. Hsp70 can originate from dying cells as a free protein, and it may be actively released in lipid microvesicles, such as exosomes, by viable malignantly transformed cells (DeMaio 2011; Gastpar et al. 2005; Pockley et al. 2014; Cordonnier et al. 2017). High intracellular Hsp70 levels in human tumors are associated with elevated Hsp70 levels in the circulation (Gunther et al. 2015). The purpose of this study was to examine whether extracellular Hsp70, as determined by the lipHsp70 ELISA, could also serve as a useful tool for tumor detection in canine patients. Studies with domestic dogs as model organisms are of great value for translational medicine, because dogs cohabitate with their owners in the same (clean but not completely germ-free) environment, are kept until reaching an old age, receive a high level of healthcare, show a high genetic diversity, and spontaneously develop tumors in the same way as humans (Rowell et al. 2011; Grosse et al. 2014). All pet dogs included in this study developed spontaneous tumors of different entities.

Compared with humans (Breuninger et al. 2014), the mean Hsp70 serum values of dogs were slightly lower. No significant correlations of the Hsp70 serum concentrations were found regarding weight, age, gender, breed, and pedigree of the dogs. Although Hsp70 levels did not differ significantly in humans, plasma Hsp70 levels were always slightly lower than in serum. In dogs, Hsp70 levels in the plasma were significantly lower compared with those of serum. The reasons for these differences have not yet been elucidated. One might speculate that due to generally lower Hsp70 levels in dogs, the negative effect of the anti-coagulant in plasma might be more pronounced in dog plasma samples compared with human plasma samples.

As shown for human serum and plasma samples, up to three repeated freezing/thawing cycles of the canine sera did not significantly impact the Hsp70 concentrations as assessed by the lipHsp70 ELISA. However, due to problems in protein stability, more than three freezing/thawing cycles of the serum resulted in significantly lower Hsp70 concentrations in humans and dogs. As previously shown by our group, Hsp70 tended to spontaneously form self-aggregates (Stangl et al. 2018) that could negatively affect the results of the lipHsp70 ELISA due to its oligomerization domain.

As already demonstrated for human blood samples, a massive food intake (one-third of the daily ration) before blood donation did not influence the Hsp70 levels measured with the lipHsp70 ELISA. This was an important prerequisite for medical studies in pet animals.

To the best of our knowledge, Hsp70 serum concentrations have never been investigated in dogs as a tumor biomarker. Our study showed that circulating Hsp70 levels were higher in tumor-bearing dogs compared with control animals. Especially round cell tumors, a heterogeneous group of tumors consisting of hematological malignancies, including mast cells, plasma cells, lymphocytes, histiocytes, and melanomas (Raskin and Meyer 2016) exhibited significantly elevated Hsp70 serum levels. Therefore, we proposed circulating Hsp70 as a potential diagnostic tumor biomarker for dogs with round cell tumors. A sub-classification of this tumor type revealed the highest Hsp70 concentrations in dogs with mast cell tumors. Hsp70 serum concentrations in dogs with melanoma were also found to be elevated compared with healthy control animals, but the data were not statistically significant due to the relatively low number of animals.

Canine patients with mesenchymal and epithelial tumors did not show significantly increased Hsp70 serum concentrations compared with healthy animals. Decreased intracellular Hsp70 concentrations were very unlikely to explain this finding, because Western blot analysis showed an equally strong Hsp70 expression in canine round cell and mesenchymal tumor cell lines. Therefore, we speculated that round cell tumors could have a higher capacity to release Hsp70, as a free molecule or in exosomes, into the bloodstream compared with solid mesenchymal or solid epithelial tumors. Differences in the total tumor burden could also provide an explanation for the lower Hsp70 concentrations in the circulation of these tumor entities. In tumor mouse models (Bayer et al. 2014) as well as in human patients with NSCLC (Gunther et al. 2015), the tumor volume correlated with serum Hsp70 levels. However, this question was not addressed in the present study as the tumor volumes of the dogs were not determined. Future studies with larger cohorts of canine patients and different tumor volumes are necessary to define the minimal volume of tumors that is detectable by elevated Hsp70 serum levels in dogs. Furthermore, kinetic measurements of the Hsp70 serum levels may also be useful for monitoring therapy outcome by minimally invasive methods in canine patients.
